# Ectomycorrhizal fungal assemblages of *Abies alba* Mill. outside its native range in Poland

**DOI:** 10.1007/s00572-015-0646-3

**Published:** 2015-06-14

**Authors:** Maria Rudawska, Marcin Pietras, Iwona Smutek, Paweł Strzeliński, Tomasz Leski

**Affiliations:** Laboratory of Symbiotic Associations, Institute of Dendrology of the Polish Academy of Sciences, Parkowa 5, 62-035 Kórnik, Poland; Department of Forest Management, University of Life Sciences in Poznań, Wojska Polskiego 71C, 60-625 Poznań, Poland

**Keywords:** Silver fir, Symbiotic fungi, Ectomycorrhizal diversity, Host-generalist, *Cenococcum geophilum*

## Abstract

*Abies alba* (Mill.) is an important forest tree species, native to the mountainous regions of Europe but has been also widely introduced in the lowlands outside its native range. Like most forest tree species, *A. alba* forms obligate mutualisms with ectomycorrhizal (ECM) fungi. This investigation sought to examine ECM fungal communities of *A. alba* when the species grows 400 km north of its native range in the region of Pomerania in Poland. We surveyed for ECM fungi by sampling live roots from four mature forest stands where the *A. alba* component ranged from 20 to 100 %. Ectomycorrhizal fungal symbionts were identified based on morphotyping and sequencing of the internal transcribed spacer (ITS) of nuclear ribosomal DNA (rDNA). Thirty-five ECM fungal taxa were distinguished on root tips of *A. alba* from all tested stands with 22 to 27 ECM fungal taxa in the individual stand. The diversity and similarity metrics revealed a lack of statistical differences in the structure of the ECM fungal community between stands varying in overstory tree composition. *Cenococcum geophilum* was the most common fungal species at all investigated *A. alba* stands, with an abundance of 50 to 70 %. The ECM community was characterized by the lack of *Abies-*specific fungal symbionts and a rich and diverse suite of host-generalist mycobionts that seem to be sufficient for successful growth and development of *A. alba* outside of its native range.

## Introduction

Forest tree species may occur outside their historic natural range in response to climate change and global warming or because humans have deliberately introduced a tree species in a region where the species is very productive. The latter occurred with *Abies alba* Mill. (silver fir) in the lowlands of northern Poland (Pomerania) where this European mountain tree species found excellent conditions for growth and development 400 km north of its native range (Bijak 2010). The history of introduction of *A. alba* in the area of Polish Pomerania dates back to the end of the nineteenth century when experimental stands were established by German and Austrian foresters Schwappach, Wiedemann, and Cieślar (Bellon et al. 1977) and exist to the present time. These stands are still growing very well, and several productivity indicators (mean tree height, crown characteristics, tree-ring widths, health status, high abundance of natural regeneration) are similar to those obtained for *A. alba* grown in the native range of this tree species in southern Poland (Bijak 2010).

Within its native region, like most temperate and boreal tree species, *A. alba* develops obligate mutualisms with ectomycorrhizal (ECM) fungi, and this symbiosis plays a significant role in the survival and growth of trees (Smith and Read 2008). In general, *A. alba* has received little attention as an ECM host. Many studies on ECM fungi accompanying *A. alba* tend to focus on surveys of fungal fruiting bodies. In most other cases, ectomycorrhizas of *A. alba* were only characterized as “mycorrhizal types” without detailed identification of the fungal partner (Comandini et al. 2001 and references therein). *A. alba* ectomycorrhizas are also highly underrepresented in “An Information System for Characterization and DEtermination of EctoMYcorrhizae” (DEEMY), the largest online information system for morphological and anatomical determination of ectomycorrhizas (Agerer and Rambold 2004–2014). In this database comprising a total of 554 items, only four descriptions with taxonomic names of *A. alba* ectomycorrhizas are presented (*Lactarius intermedius* Krombh. ex Berk. & Broome, *Lactarius salmonicolor* R. Heim & Leclair, *Lactarius subsericatus* Kühner & Romagn., and *Tricholoma bufonium* (Pers.) Gillet). Still, less has been published about the ECM fungal symbionts that associate with *A. alba* based on molecular approaches (Eberhardt et al. 2000; Schirkonyer et al. 2013; Ważny 2014). As ECM symbiosis is required for the good growth and survival of many important forest tree species (Smith and Read 2008), the success of a tree species outside its natural range involves among other factors the availability of compatible symbiotic partners (Pringle et al. 2009; Vellinga et al. 2009; Dickie et al. 2010). Recently, several authors have intensively studied this issue in terms of different exotic tree species (e.g., Walbert et al. 2010; Bahram et al. 2013; O’Hanlon and Harrington 2012; Trocha et al. 2012; O’Hanlon et al. 2013; Lothamer et al. 2014). However, essentially, no data are available related to ECM fungal assemblages of *A. alba* when the tree species is growing outside its natural range. To fill gaps in this area of research, we surveyed for ECM fungi by sampling live roots from four mature forest stands of *A. alba* from the Polish Pomerania region where the fir component ranged from 20 to 100 %. *A. alba* was introduced ca. 100 years ago using seeds of unknown provenance; however, recent analyses of chloroplast microsatellites showed that the Sudety Mts. are the most probable region of origin of these fir trees (Dzialuk et al. 2013). Because suitable generalist ECM fungi are known to occur in this region (Stasińska 1999; Wojewoda 2003), and the introduced fir trees are exhibiting excellent growth rates, we hypothesized that the overall diversity of the ECM fungal community would be within the wide range of taxa reported from other coniferous forests. This assumption is supported by the finding that the non-native ECM trees often accept local mycorrhizal fungi as their symbiotic partners (Cullings et al. 2000; Tedersoo et al. 2007; Bahram et al. 2013; O’Hanlon et al. 2013; Kohout et al. 2011). Such overlap in compatibility with the local native ECM fungi (Watling 1995) likely appears in our studies, because *A. alba* and co-occurring trees from tested stands (*Picea abies*, *Pinus sylve*stris, *Fagus sylvatica*, *Quercus* sp.) come from temperate forest families that overlap in their native geographical ranges. A similar scenario was recently suggested and confirmed when non-native *Pinus* was introduced into forested areas of Iran (Bahram et al. 2013). Because *A. alba* trees have been introduced to the Pomerania region as seed without any fungal inoculum, and due to limitations of fungal dispersal (Galante et al. 2011; Horton et al. 2013) we further hypothesized that the ECM community of introduced trees will lack host-specific fungal symbionts. Results obtained will greatly enhance our understanding about the ECM fungal communities of *A. alba* when this tree species is growing outside its natural range.

## Materials and methods

The study was conducted 400 km north of the natural range of *A. alba* in the central and eastern parts of Polish Pomerania (northern Poland) at four discreet forest stands, situated in the forest districts of Kartuzy (K), Lipusz (L), Osusznica (O), and Sławno (S) separated by at least 30 km. Selected stands were established after clear-cutting of managed mixed forests in the late nineteenth century and early twentieth century (Strzeliński, unpublished data). All stands had similar climatic and soil conditions with a mean annual temperature of +8 °C, ranging between −0.4 °C in January and 18.5 °C in July and a mean annual precipitation of about 745 mm. Details about stands characteristics are presented in Table 1.

**Table 1:** Location, soil characteristics, plant association, and tree species composition of the study stands

	Stand			
	K	L	O	S
Geographic coordinates	54° 13′ 27.2″ N, 17° 59′ 47.9″ E	54° 14′ 25.0″ N, 17° 47′ 43.3″ E	54° 06′ 58.8″ N, 17° 23′ 35.6″ E	54° 29′ 02.8″ N, 16° 37′ 00.4″ E
Soil type	Brown acid	Brown acid	Luvisol	Brown acid
Humus form	Mull	Moder	Moder-mull	Moder-mor
Thickness of organic layer (cm)	20	20	19	22
pH _H2O_ O-horizon	3.9	3.9	3.9	4.0
pH _H2O_ A-horizon	4.0	4.6	4.6	4.3
Plant association^a^	*Fago-Quercetum*	*Abies alba*-*Deschampsia flexuosa*/*Luzulo pilosae-Fagetum*	*Luzulo pilosae-Fagetum*	*Luzulo pilosae-Fagetum*
% Tree species composition and tree age (years)	40 *Abies* (106) 40 *Fagus* (106) 10 *Pinus* (106) 10 *Quercus* (106)	50 *Picea* (92) 40 *Abies* (92) 10 *Fagus* (162)	100 *Abies* (115)	60 *Fagus* (105) 20 *Abies* (105) 10 *Picea* (105) 10 *Pinus* (105)

*K* Kartuzy, *L* Lipusz, *O* Osusznica, *S* Sławno, *Abies Abies alba*, *Fagus Fagus sylvatica*, *Picea Picea abies Pinus Pinus sylvestris*, *Quercus Quercus* spp.

^a^According to Matuszkiewicz 2001

Soil samples for mycorrhizal assessment were gathered in October 2012. A small spade was used to collect a 15 × 15 × 15 cm soil cube, starting from the organic layer after the removal of the litter layer. Sampling was done at least 30 m from the stand boundary to avoid an ecotone effect on the ECM fungal communities, and samples were separated by at least 6 m to avoid spatial autocorrelation. In each stand, 16 soil samples were randomly collected, from across each of four stands yielding a total of 64 samples. One non-random aspect of sampling was that samples were collected at about 50–80 cm from an *A. alba* tree trunk to ensure that we were sampling *A. alba* and no other tree sp. roots. Soil cores were secured by placing them into labeled individual ziplock plastic bags and transporting to the laboratory at the Institute of Dendrology, Kórnik, Poland. All samples were stored at 4 °C before being processed, but were not held longer than 2 weeks. Prior to analysis, soil samples were soaked in water at 4 °C overnight, and then, fine roots (<2 mm) were separated and cleaned from the soil in a sieve under cold running water. Fine roots with ectomycorrhizas were cut into 4–5-cm fragments. For each soil sample, three random root subsamples were counted until each subsample had approximately 250 root tips. Discrimination between ectomycorrhizas of *Abies* and ectomycorrhizas of other tree species, potentially present in the soil samples, was based on differences in morphology and the diameter of unramified mycorrhizal tips of *Abies* in relation to ectomycorrhizas of spruce, pine, or some deciduous trees. Diameter of *Abies* mycorrhizas is about 30 % larger than that of *Pinus* or *Picea* ectomycorrhizas and 50–60 % larger than that of ectomycorrhizas of broadleaf trees. Examination of the ectomycorrhizas was performed under a dissecting microscope at ×10 to ×100 magnification (SteREO Discovery V12, Zeiss). The ectomycorrhizas were described according to Agerer (1987–2008) based on macroscopic features (ramification system; color, shape, texture, and thickness of the mantle; presence and organization of the emanating hyphae; rhizomorphs; and other elements) and classified into separate morphotypes. The number of ectomycorrhizas of each morphotype and ECM colonization ratio were noted separately for each sample. For each ECM morphotype, five to 10 carefully cleaned ECM tips were placed in Eppendorf tubes in Milli-Q water and frozen at −20 °C until processing for molecular analysis. Throughout molecular identification, each morphotype sample was processed separately. Specimens were combined for abundance calculation only after the DNA analysis showed that morphotypes were the same. The relative abundance of each identified morphotype was calculated as a percentage of the total number of ectomycorrhizas of each subsample; then, data were averaged in each sample and in overall samples from each study stand. Ectomycorrhizal fungal symbionts were identified based on PCR amplification (wit primer pair ITS-1f and ITS-4) and sequencing of the internal transcribed spacer (ITS) of nuclear ribosomal DNA (rDNA). Total DNA was extracted from frozen ectomycorrhizas with the miniprep method developed by Gardes and Bruns (1996) or using a GeneMATRIX Plant & Fungi DNA Purification Kit (EURx Ltd., Gdańsk, Poland). Multiple PCR products (double DNA bands on the gel) were omitted from the analysis. Species-level identification of mycorrhizas was defined as the sharing of >98 % ITS region sequence identity with the reference sequence obtained from sporocarp vouchers, over a length of at least 450 bp. All fungal species names were updated to their current nomenclature using the online resources Index Fungorum (http://www.indexfungorum.org as of the date 07.04.2015). Selected sequences have been deposited in GenBank under the accession numbers KP230465-230499. Complete methods for molecular analysis and identification of ECM morphotypes are presented in our previous papers (Leski et al. 2010; Leski and Rudawska 2012).

The belowground ECM fungal diversity at each study stand was expressed as the number of recognized ECM taxa (taxa richness) and evaluated using Shannon–Wiener diversity and Simpson’s dominance indices. Jackknife 2 richness estimator was calculated with EstimateS 8.0 software (Colwell 2006), using 1000 randomized runs with sample replacement.

Multivariate analyses of the belowground community of ECM fungi were performed using PAST 2.13 software (Hammer et al. 2001), with the Bray–Curtis dissimilarity coefficient, based on square root-transformed data. The data matrix consisted of 64 samples with the relative abundance of each ECM fungal taxon within each sample. Nonmetric multidimensional scaling ordination (NMDS) was used to illustrate differences (based on the Bray–Curtis matrix) between ECM fungal communities from the investigated stands. Differences in relative abundance of ECM fungal taxa between study stands were tested with one-way analysis of similarity (ANOSIM). Analysis of variance with Tukey’s test was used to compare the mean taxa richness and ecological indices on a sample basis between tested *A. alba* stands.

## Results

The examination of fine roots of *A. alba* from all stands revealed nearly 100 % ECM colonization. Morphological assessment was carried out on 35,825 ECM tips from 64 samples (16 samples per stand). Based on gross morphology, 38 mycorrhizal morphotypes were distinguished in all study stands. Overall, 221 out of the 248 ECM tips representing all morphotypes yielded PCR products and were subjected to ITS sequence analysis. The total successful amplification rate was 89 %. Based on sequencing of ITS fungal rDNA, 35 mycorrhizal fungal taxa were identified. Of these 35 ECM fungal taxa, 28 were assigned to a species level and seven to genus (Table 2). The taxa richness of mycorrhizal fungi ranged from 27 taxa (stand S) and 26 taxa (stand L) to 23 (stand O) and 22 taxa (stand K). Average taxa richness noted in soil samples ranged between 9.6 and 12.4, without significant differences between stands according to ANOVA (Table 2).

**Table 2:** Molecular identification, relative abundance (±SE), observed total and mean species richness (±SE), estimated species richness, and ecological indices for ectomycorrhizal fungi on the roots of *Abies alba* trees from stands K, L, O, and S

Identification	Accession	Closest match	Identity (%)	Relative abundance (%)—stand			
				K	L	O	S
*Cenococcum geophilum* Fr.	KP230465	*Cenococcum geophilum* (JN943885)	99	68.0 ± 10.1	50.2 ± 6.2	69.8 ± 6.2	55.9 ± 6.0
*Tomentella stuposa* (Link) Stalpers	KP230466	*Tomentella stuposa* (UDB002429)	100	7.4 ± 1.2	19.1 ± 1.6	7.5 ± 0.9	14.9 ± 2.1
*Lactarius camphoratus* (Bull.) Fr.	KP230467	*Lactarius camphoratus* (KF432971)	100	3.5 ± 0.2	1.2 ± 0.1	2.0 ± 0.1	0.5 ± 0.1
*Russula fellea* (Fr.) Fr.	KP230468	*Russula fellea* (UDB000110)	99	3.04 ± 0.3	1.0 ± 0.1		1.5 ± 0.1
*Byssocorticium atrovirens* (Fr.) Bondartsev & Singer	KP230469	*Byssocorticium atrovirens* (UDB000075)	99	2.8 ± 0.2	0.1 ± 0.1		0.1 ± 0.1
*Tomentella albomarginata* (Bourdot & Galzin) M.P. Christ.	KP230470	*Tomentella albomarginata* (UDB018851)	100	2.4 ± 0.3	3.4 ± 0.3	1.9 ± 0.2	4.2 ± 0.2
*Tomentella terrestris* (Berk. & Broome) M.J. Larsen	KP230471	*Tomentella terrestris* (UDB011638)	100	2.4 ± 0.4	1.2 ± 0.2	1.2 ± 0.2	1.8 ± 0.1
*Cantharellus* sp.	KP230472	*Cantharellus tubaeformis* (UDB002389)	96	2.0 ± 0.5	2.6 ± 0.3	1.2 ± 0.2	2.0 ± 0.2
*Xerocomellus pruinatus* (Fr. & Hök) Šutara	KP230473	*Xerocomus pruinatus* (UDB000481)	99	1.5 ± 0.3	1.7 ± 0.3	0.9 ± 0.2	2.0 ± 0.1
*Genea* sp.	KP230474	*Genea hispidula* (AJ969623)	96	1.3 ± 0.2			0.2 ± 0.1
*Cortinarius malachius* (Fr.) Fr.	KP230475	*Cortinarius malachius* (JQ888160)	99	1.2 ± 0.4	2.6 ± 0.4	4.2 ± 0.9	1.9 ± 0.2
*Inocybe geophylla* (Bull.) P. Kumm.	KP230476	*Inocybe geophylla* (JQ888171)	98	1.2 ± 0.5	1.7 ± 0.5	0.5 ± 0.1	1.5 ± 0.4
*Cortinarius* sp. 2	KP230477	*Cortinarius camphoratus* (UDB011341)	95	0.9 ± 0.1	1.1 ± 0.3	0.8 ± 0.3	0.3 ± 0.1
*Geopora cervina* (Velen.) T. Schumach.	KP230478	*Geopora cervina* (UDB016155)	94	0.7 ± 0.2	1.4 ± 0.4	0.1 ± 0.1	0.6 ± 0.3
*Cortinarius fulvescens* Fr.	KP230479	*Cortinarius fulvescens* (HQ604731)	99	0.6 ± 0.4	0.9 ± 0.2	0.3 ± 0.1	0.8 ± 0.4
*Meliniomyces variabilis* Hambl. & Sigler	KP230480	*Meliniomyces variabilis* (HQ157931)	99	0.4 ± 0.2	1.0 ± 0.3	0.6 ± 0.3	0.4 ± 0.2
*Lactarius aurantiacus* (Pers.) Gray	KP230481	*Lactarius aurantiacus* (KF432974)	99	0.4 ± 0.2	1.8 ± 0.6		0.5 ± 0.3
*Lactarius rufus* (Scop.) Fr.	KP230482	*Lactarius rufus* (KF241543)	99	0.1 ± 0.2		>0.1	2.0 ± 1.1
*Pseudotomentella tristis* (P. Karst.) M.J. Larsen	KP230483	*Pseudotomentella tristis* (UDB000032)	100	0.1 ± 0.1	3.6 ± 1.1	0.5 ± 0.3	0.3 ± 0.1
*Tylopilus felleus* (Bull.) P. Karst.	KP230484	*Tylopilus felleus* (HM190016)	100	>0.1	0.2 ± 0.1	0.1 ± 0.2	
*Peziza* sp.	KP230485	*Peziza succos*a (UDB015317)	97	>0.1	0.9 ± 0.3	0.5 ± 0.2	1.6 ± 0.8
*Laccaria laccata* (Scop.) Cooke	KP230486	*Laccaria laccata* (UDB015789)	96	>0.1			
*Piloderma fallax* (Lib.) Stalpers	KP230487	*Piloderma fallax* (UDB001614)	99		2.6 ± 0.6	4.1 ± 0.9	3.1 ± 0.6
*Tylospora asterophora* (Bonord.) Donk	KP230488	*Tylospora asterophora* (UDB002469)	100		0.6 ± 0.2		
*Tomentella botryoides* (Schwein.) Bourdot & Galzin	KP230489	*Tomentella botryoides* (UDB000258)	100		0.6 ± 0.2		
*Craterellus lutescens* (Fr.) Fr.	KP230490	*Craterellus lutescens* (UDB019796)	96		0.3 ± 0.1	0.4 ± 0.2	0.8 ± 0.4
*Amanita muscaria* (L.) Lam.	KP230491	*Amanita muscaria* (AB080984)	99		0.1 ± 0.1	1.6 ± 0.3	0.2 ± 0.2
*Imleria badia* (Fr.) Vizzini	KP230492	*Xerocomus badius* (HQ207696)	100		0.1 ± 0.3	0.4 ± 0.2	
*Tuber puberulum* Berk. & Broome	KP230493	*Tuber puberulum* (HM190013)	99		>0.1		
*Cortinarius* sp. 1	KP230494	*Cortinarius traganus* (UDB011350)	97			0.2 ± 0.1	
*Russula* sp. 1	KP230495	*Russula paludosa* (JF908659)	97			0.1 ± 0.1	
*Russula* sp. 2	KP230496	*Russula xerampelina* (UDB002533)	95				1.1 ± 0.3
*Russula ochroleuca* Fr.	KP230497	*Russula ochroleuca* (JF908647)	100				0.7 ± 0.2
*Cortinarius semisanguineus* (Fr.) Gillet	KP230498	*Cortinarius semisanguineus* (UDB001553)	100				0.6 ± 0.3
*Laccaria amethystina* Cooke	KP230499	*Laccaria amethystina* (HM189776)	100				0.1 ± 0.2
Abundance of shared species				92.4	92.6	91.9	88.6
Abundance of stand characteristic species				>0.1	1.2	0.2	2.5
Taxa richness				22	26	23	27
No. of stand characteristic taxa				1	3	2	4
Average taxa richness per sample				9.62 ± 1.51	10.87 ± 2.64	10.62 ± 3.58	12.37 ± 1.41
Jackknife 2				31.76	33.12	27.81	31.64
Shannon diversity				1.12 ± 0.51	1.31 ± 0.46	1.02 ± 0.42	1.40 ± 0.42
Simpsons dominance				0.51 ± 0.19	0.42 ± 0.15	0.55 ± 0.18	0.41 ± 0.17

*K* Kartuzy, *L* Lipusz, *O* Osusznica, *S* Sławno, *abundance of shared species* the abundance of species shared with at least one other site, *abundance of the stand characteristic species* abundance of species present in only one stand

The fungi identified were Basidiomycetes (orders: *Agaricales*, *Atheliales*, *Boletales*, *Thelephorales*, *Russulales*, and *Cantharellales*) and Ascomycetes (orders: *Pezizales*, *Helotiales*, and *Mytilinidiales*). In terms of taxa richness, the investigated ECM fungal communities were dominated by representatives of the orders *Agaricales* (nine taxa), *Russulales* (seven taxa), *Thelephorales* (five taxa), and *Pezizales* (four taxa). Fifteen fungal taxa were common in all study stands. One taxon was found exclusively in stand K, two in stand O, and three in stand L, and four taxa were restricted to stand S. The relative abundance of fungal taxa that were specific for the individual stand ranged from <0.1 % in stand K to 2.5 % in stand S (Table 2).

Irrespective of the stand, the ECM fungal communities were dominated by *Cenococcum geophilum* (relative abundance from 50.2 to 69.8 %). The second most abundant taxon was *Tomentella sublilacina* (relative abundance 7.4–19.1 %). Other species always occurred with a relative abundance of less than 5 % (Table 2).

**Fig. 1:**
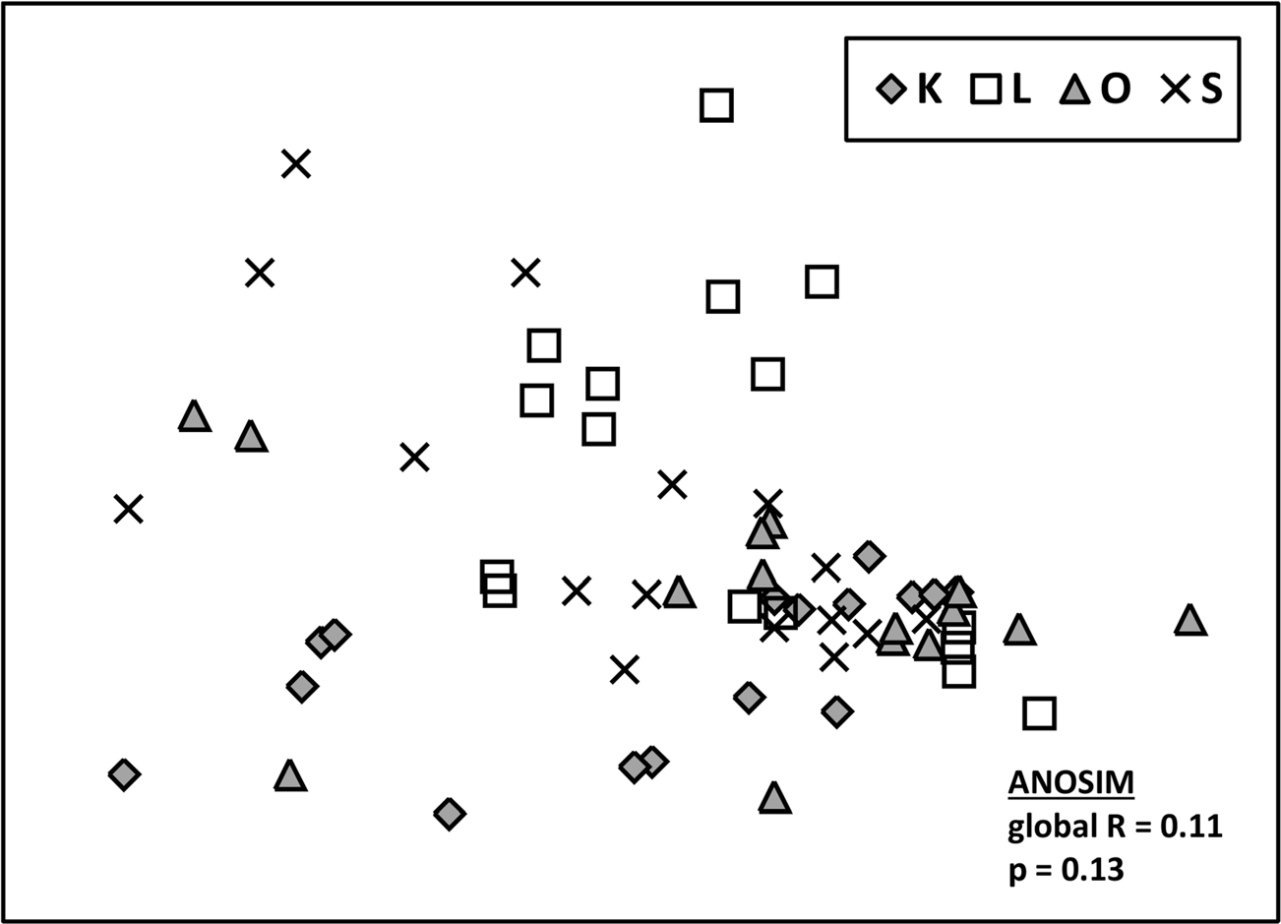
Nonmetric multidimensional scaling (NMDS) ordination of ectomycorrhizal fungal communities of *Abies alba* trees from stands K, L, O, and S (NMDS parameters: *R* ^2^ of the axis 1 = 0.49, *R* ^2^ of the axis 2 = 0.37, final stress = 0.16)

The Shannon’s diversity and Simpson dominance indices for the ECM assemblages of tested *A. alba* did not differ significantly between study stands (Table 2). ANOSIM and NMDS analyses were used to assess the influence of study stands on ECM fungal communities. Based on ANOSIM analysis, fungal communities were not significantly different between study stands (global *R* = 0.11; *P* = 0.13). Consistently, the NMDS ordinations of the ECM fungal communities (final stress = 0.16) showed no clear grouping of samples according to stand identity (Fig. 1).

## Discussion

The overall species richness observed in our study support our assumption that the diversity of ECM fungal community of *A. alba* falls within the broad range of variability described in sequencing studies of other coniferous trees grown in their native temperate range. Examples include studies of Scots pine (29 taxa, Rudawska et al. 2011; 43 taxa, Pickles et al. 2012), Norway spruce (16 taxa, Kjøller et al. 2012), and Douglas fir (31–45 taxa depending on the site, Kranabetter et al. 2012). The 35 ECM fungal taxa characterized on root tips of *A. alba* from four mature forest stands correspond to 81 % of the potential estimated species richness of 44 taxa. Given the sampling strategy of our study, such level of discovered fungal species richness in relation to estimated richness may indicate that ECM symbionts compatible with *A. alba* outside of its native range have a rather homogenous spatial distribution. Moreover, low variation in relative abundances of the prevailing ECM fungal species further confirmed that certain fungal species were similarly abundant across the stands.

Comparison of our observed species richness with other data on ECM fungus community structure carried out directly in natural *A. alba* stands is difficult due to the scarcity of data that is mostly restricted to aboveground ECM fungal sporocarp diversity. Depending on the number of seasons and locations surveyed, Laganà et al. (2000, 2002) observed 43 to 61 ECM fungal species in *A. alba* monoculture forests in Tuscany, Italy. In contrast, aboveground ECM fungal diversity of *A. alba* grown outside its natural range in Mecklenburg-Western Pomerania, Germany, was much lower and amounted to only 22 species of ECM fungi (Unterseher et al. 2012). Belowground studies of the ECM fungal assemblages that used morphological characterization of ectomycorrhizas revealed 25 to 48 taxa on *A. alba* trees grown in the mountainous natural woods of the Abruzzo region, Italy (Comandini et al. 1998, 2001). However, resolution of ECM fungal diversity based on morphotyping is often low and only rarely allows for an unambiguous identification of mycorrhizas at the fungal species level (Sakakibara et al. 2002). Studies of the ECM fungal communities associated with *A. alba* using direct sequencing of the ECM root tip DNA are scarce compared with similar studies of other coniferous and deciduous trees (Comandini et al. 2012). Only 15 mycorrhizal taxa were identified with molecular methods in 100-year-old pure stands of *A. alba* in the Taunus Mountains in Central Germany (Schirkonyer et al. 2013) where *A. alba* is not native. The mycorrhizal species richness was much lower in that study than ours and was likely underestimated due to the low sampling effort of that research (only 46 mycorrhizal tips were analyzed). The only comprehensive sequencing data on the ECM fungal community of *A. alba* are from studies conducted on seedlings naturally regenerating under Silver fir and Scots pine canopy in the Polish Carpathians (Ważny 2014). Overall, ECM fungal richness in those stands was higher than the richness obtained in our studies, with a total of 49 ECM fungal taxa reported. Part of the discrepancy in overall species richness between the study in the Polish Carpathians and our studies in Polish Pomerania is likely due to sampling intensity (six vs four sites and 30 vs 16 samples per site, respectively). However, at the individual stand level, species richness was higher in our study ranging from 22 to 27 ECM fungal taxa, depending on the stand and canopy tree composition. Taken together, we believe that the ECM fungus suite of *A. alba* when grown outside the natural range is not restricted on a per stand basis, and *A. alba* trees are readily colonized by available and compatible ECM fungi.

It is generally accepted that the co-occurrence of different host tree species within a stand promotes ECM fungus diversity at the local scale (Ishida et al. 2007; Morris et al. 2008; Tedersoo et al. 2008). Therefore, we expected that that presence of closely related confamilials (*P. abies* and/or *P. sylvestris*) and more distantly related non-confamilials (*Quercus* spp. and/or *F. sylvatica*) in tested stands would affect ECM fungal species richness of *A. alba* trees. However, in our studies, the resulting diversity and similarity metrics revealed a lack of statistical differences in the structure of the ECM fungal community between stands that varied in overstory tree composition. This indicates that neighboring trees of different species had no influence on the ECM fungal community of *A. alba*. Interestingly, even the pure stand of *A. alba* had a similar ECM fungal community structure to stands with high overstory tree composition. This confirms previous findings that in the northern hemisphere where the range of many forest tree species overlap greatly, fine roots of non-native species can be colonized by indigenous ECM fungi (Trocha et al. 2012).

It has been well documented that moving different ECM tree species within/between the temperate and boreal regions (east–west movement) rarely presents problems for suitable indigenous ECM fungi to colonize the introduced tree species (Buée et al. 2011; O’Hanlon et al. 2013). In contrast, in north–south movements of ECM tree species, especially over thousands of kilometers (e.g., across the equator), situations can arise wherein the ECM fungal community is not suitable for ECM tree success (Dickie et al. 2010; Nuñez et al. 2009). Because both the direction and magnitude of *A. alba* movement in our study were small, we found suitable ECM fungi present at the destination site.

The ECM fungal taxa identified within all study stands are among the more common members of ECM fungal communities of temperate and boreal forests in Europe, including *C. geophilum* and members of the genera *Tomentella*, *Lactarius*, *Russula*, *Cortinarius*, and *Laccaria. C. geophilum* and many members of these genera exhibit broad host ranges and allow potential development of mycelial networks between co-occurring trees (Kennedy et al. 2003; Nara 2006). These taxa have been previously reported from mycocoenological studies from different regions of Poland (Wojewoda 2003).

Among the 35 ECM fungal species found in our study, only 10 species, including *C. geophilum*, *Tomentella stuposa*, *Xerocomellus pruinatus*, *Lactarius aurantiacus*, *Piloderma fallax*, *Tylospora asterophora*, *Amanita muscaria*, *Imleria badia*, *Tuber puberulum*, and *Laccaria amethystina*, were also common associates on *A. alba* seedlings grown in its native range (Ważny 2014). These 10 ECM fungal species colonized approximately 80 % of the root tips on mature *A. alba* trees outside of its native range, but only 25 % within its native range in the study of Ważny (2014). Contrary to the findings of Ważny (2014), we did not observe fungal symbionts belonging to the genera *Amphinema*, *Clavulina*, *Elaphomyces*, *Hydnotrya*, *Hydnum*, or *Sebacina*. Such discrepancy in relative abundance of shared species and species composition between ECM fungal communities of *A. alba* trees inside and outside its native range demonstrates the importance of site characteristics (pH, litter and soil quality, climate, etc.) in structuring ECM community (Jumpponen and Egerton-Warburton 2005).

Along with the occurrence of generalist fungal species as a component of ECM colonization of *A. alba*, we did not observe *Abies*-specific symbionts, such as *L. salmonicolor*, which is widespread in above- and belowground communities in the natural range of *A. alba* (Comandini et al. 1998, 2001; Laganà et al. 2000; Ważny 2014). Other fungal species that exhibited some level of *Abies* preference (*Lactarius albocarneus* Britzelm., *L. intermedius*, *Russula cavipes* Britzelm.) were also absent in our studies and in other surveys of *A. alba* grown outside its natural range (Unterseher et al. 2012; Schirkonyer et al. 2013). The absence of the fungal symbionts that show preference for *Abies* may be ascribed to several factors: (1) soil and environmental conditions are inappropriate for the growth and development of the mycelium of host-specific fungal symbionts; (2) geographic distance from the natural range of *A. alba* negatively influences the dispersal and competitive abilities of fungal propagules; and (3) sampling design of our studies was insufficient to detect the ECM host-specific symbionts, known to occur with low abundance (Ważny 2014). Our data suggest *A. alba* does not extensively rely on host-specific fungi, a feature characteristic of many late successional tree species; this supports the suggestion of Kropp and Trappe (1982) that was further confirmed by Horton et al. (2005), that selection pressure in later successional tree species pushes many species away from host specificity.

*C. geophilum* was the most common species at all investigated *A. alba* stands with an abundance of 50–70 %. Such abundance of *C. geophilum* might be somewhat overestimated due to the robust and melanized mantles of this fungus that allow the *C. geophilum* ectomycorrhizas to persist 4–10 times longer than ectomycorrhizas of other ECM fungal species (Fernandez et al. 2013). This unique feature makes morphological determination of this fungus possible even after the ectomycorrhizas are dead (Valentine et al. 2004). But even knowing that only 40 % of *C. geophilum* ectomycorrhizas are vital in autumn (Fernandez et al. 2013), this species remains the most abundant component of *A. alba* ECM fungal communities. An important factor that may contribute to the predominance of *C. geophilum* might be the thick organic layer on the forest floors of the tested stands. This layer is characterized by high fluctuations of soil temperature and moisture content, often to the point of drying. *C. geophilum* is well known as a very competitive fungus that grows very well in such conditions (Hasselquist et al. 2005; Matsuda et al. 2009; Dickie 2007).

It is generally recognized that tree species growing outside of their native range harbor relatively species-poor ECM communities (Tedersoo et al. 2007; Nuñez et al. 2009; Dickie et al. 2010; Walbert et al. 2010) because of the low number of compatible symbiotic fungi, dispersal limitations, and a frequent lack of host-specific symbionts. We did not observe evident impoverishment of ECM fungal assemblages during our study of *A. alba* stands in the Pomerania region when compared with the ECM constituents inside the native range (Ważny 2014). The ECM community was characterized by a lack of *Abies-*specific fungal symbionts and a rich and diverse suite of local host-generalist mycobionts. However, there are some limitations in our sampling strategy that are important to note. Due to time constraints, we only sampled at one point in time, so our findings cannot represent the year-round fungal community. In terms of vertical variation in the soil profile, sampling was limited to 15-cm depth; thus, some taxa with the preference to mineral soil horizons could be left undetected. Horizontal variation may also play a part in our study system, but in this case, we tried to avoid spatial autocorrelation between soil cores by sampling more than 6 m apart. Recently, the problem of horizontal, vertical, and temporal variations in ECM communities has been thoroughly reviewed (Bahram et al. 2014; O’Hanlon 2012) and should be carefully considered when planning future experiments.
